# Associations of the Baseline Level and Change in Triglyceride Glucose‐Body Roundness Index With Cardiovascular Diseases: Two Large Prospective Cohorts in Europe and Asia

**DOI:** 10.1002/kjm2.70305

**Published:** 2026-10-22

**Authors:** Wen‐Yang Han, Yi‐Qun Li, Pin‐Ni Yang, Yi‐Ming Jia, Lu‐Lu Sun, Rui‐Rui Wang, Meng‐Yao Shi, Xiao‐Wei Zheng, Yong‐Hong Zhang, Zheng‐Bao Zhu

**Affiliations:** ^1^ Department of Public Health and Preventive Medicine, Public Health Research Center, Wuxi School of Medicine Jiangnan University Wuxi Jiangsu China; ^2^ Department of Epidemiology, Jiangsu Key Laboratory of Preventive and Translational Medicine for Major Chronic Non‐Communicable Diseases, MOE Key Laboratory of Geriatric Diseases and Immunology, School of Public Health Suzhou Medical College of Soochow University Suzhou Jiangsu China

**Keywords:** CHARLS, CVD, triglyceride‐glucose index with body round index, TyG‐BRI

## Abstract

The aim of this study was to investigate the association of baseline and changes of TyG‐BRI with incident CVD based on the UK Biobank and China Health and Retirement Longitudinal Study (CHARLS). A total of 418,510 and 7923 individuals from the UK Biobank and CHARLS were included in Analysis 1 (baseline TyG‐BRI with CVD risk). In Analysis 2 (change in TyG‐BRI with CVD risk), a total of 11,682 and 4637 participants were included in Cox proportional hazard models. We found participants with the highest baseline TyG‐BRI level were associated with increased risk of incident CVD in UK Biobank (HR = 1.45, 95% CI: 1.39–1.52) and CHARLS (HR = 1.35, 95% CI: 1.13–1.61). Multivariable‐adjusted restricted cubic spline analyses presented positive linear associations between TyG‐BRI and CVD risk. Compared to persistently low levels of TyG‐BRI (Class 1), those with persistently high levels (Class 3: 1.23 (1.02–1.49) and 1.42 (1.11–1.55); Class 4: 1.48 (1.17–1.87) and 1.45 (1.16–2.28); Class 5: 2.58 (1.86–3.58) and 1.63 (1.16–2.28) for UK Biobank and CHARLS) in follow‐up were associated with increased risks of incident CVD. In conclusion, a high baseline TyG‐BRI level and different changes in TyG‐BRI level are associated with increased risks of incident CVD.

## 1 Introduction

Cardiovascular diseases (CVD) remain the leading cause of the global disease burden [1]. According to World Health Organization (WHO) data, CVDs affected approximately 17.9 million people globally in 2019, accounting for 32% of all deaths worldwide [2, 3]. In China, CVDs are responsible for 40% of all mortalities [4], placing the country among the world's highest‐burden regions for CVD [5]. Beyond direct medical expenditures, CVDs impose substantial indirect economic and social burdens stemming from diminished work productivity and impaired quality of life [5]. Despite remarkable advances in CVD diagnosis, treatment, and interventional strategies in recent years, the global CVD burden keeps escalating. This underscores the urgent need for early identification of modifiable risk factors and high‐risk populations to mitigate the attributable disease burden of CVD [6, 7].

Insulin resistance (IR) is critically involved in the pathogenesis of metabolic syndrome, atherosclerosis, and CVD risk [8, 9]. The euglycaemic insulin clamp and intravenous glucose tolerance test can achieve high accuracy for IR evaluation; however, such techniques are labor‐intensive and expensive, limiting their practical application in routine clinical practice [10]. The triglyceride‐glucose (TyG) index, calculated based on serum triglyceride (TG) and fasting blood glucose (FBG) levels, has emerged as a simple, reliable surrogate marker for IR [10]. Growing evidence from numerous studies has validated that the TyG index is a vital biomarker for predicting CVD risk and prognosis, and it is also correlated with multiple diseases and all‐cause mortality [11, 12, 13, 14, 15, 16]. Additionally, composite TyG indices combined with obesity‐related parameters (TyG‐BMI, TyG‐WC, and TyG‐WHtR) have been demonstrated to have better predictive efficacy for cardiovascular events than conventional obesity indicators and the single TyG index [17, 18]. However, compared to traditional indices like BMI, WC, and hip circumference, the body round index (BRI), which integrates height and waist circumference, has been proven to better reflect visceral fat proportions [19], and has been proved to be more strongly associated with adverse cardiometabolic risk factors than subcutaneous adipose tissue [20]. Furthermore, data from the Environment and Chronic Disease in Rural Areas of Heilongjiang, China (ECDRAHC) study verified that the TyG‐BRI index is superior to other TyG‐derived indices in predicting ischemic stroke among rural Chinese residents [21]. Collectively, these findings indicate that both the TyG index and TyG‐BRI index can serve as promising surrogate indicators of IR to assess CVD risk.

Notably, both TyG and BRI are dynamic indicators that fluctuate over time, and multiple researchers have focused on exploring the effects of longitudinal changes in TyG and BRI on CVD risk [22, 23]. Accumulating studies have further demonstrated that longitudinal alterations and cumulative changes in TyG‐BMI and TyG‐WHtR are independently associated with the risks of stroke and CVD [24, 25]. Nevertheless, no prior studies have comprehensively evaluated the relationship between TyG‐BRI and CVD risk, and it remains unclear whether dynamic changes in TyG‐BRI can predict CVD incidence. Accordingly, this prospective study aimed to investigate the associations of baseline levels and longitudinal changes in TyG‐BRI with CVD risk, using data from the UK Biobank and the China Health and Retirement Longitudinal Study (CHARLS).

## 2 Methods

### 2.1 Study Population

The UK Biobank is a population‐based cohort comprising more than half a million individuals 40–69 years of age. The recruitment of participants was conducted between 2006 and 2010, and the participants were invited to attend 1 of the 22 centers located throughout England, Scotland, and Wales for baseline assessments, including completing baseline questionnaires, providing biological samples, and undergoing physical examinations. The details of UK Biobank's methodology and objectives have been reported elsewhere [26]. The CHARLS is an ongoing nationwide cohort study that uses a multistage clustering sample method to select participants from 28 provinces within China [27]. A total of 17,708 participants were included at baseline (2011–2012, Wave 1), and were followed up every 2 years in 2013–2014 (Wave 2), in 2015–2016 (Wave 3), in 2017–2018 (Wave 4), and in 2019–2020 (Wave 5), respectively, using a face‐to‐face computer‐assisted personal interview. The details of the CHARLS data are available at the website (http://charls.pku.edu.cn/en).

In current study, the participants met the following criteria were included in the Analysis 1: (1) without CVD at baseline (defined as CVD diagnosed prior to the date of baseline assessment), (2) with available data about TyG‐BRI, and (3) with diagnosis records of whether CVD is present in follow‐up. Finally, a total of 418,510 and 7923 individuals remained for the final Analysis 1. In Analysis 2 (outcome: CVD), those who met all the following criteria were included: (1) with two assessments of TyG‐BRI and (2) without CVD before the first assessment. Finally, a total of 11,682 and 4974 participants were eligible for subsequent analysis. Supplementary Figures 1 and 2 show the flowchart of participants in UK Biobank and CHARLS.

### 2.2 Assessment of the TyG‐BRI and Change of TyG‐BRI

Blood samples were collected by the staff of the Chinese Center for Disease Control and Prevention and were almost collected after a night of fasting. Enzymatic colormetric tests were used to assess TG and FBG and the coefficient of variations between‐assay was 1.80%. The TyG index is calculated as ln [TG (mg/dL) × FBG (mg/dL)/2] [28]. The BRI was calculated using 364.2–365.5 × (1−[WC(m)/2*π*]^2^/[0.5 × height(m)]^2^)^½^ according to previously reported studies [19]. The TyG index parameters is defined as previously described TyG‐BRI index = TyG index × BRI [29]. In addition, we utilized the “cluster” and “factoextra” packages to perform K‐means cluster analysis on two assessments of TyG‐BRI [30], aiming to provide a basis for grouping study subjects. By applying the elbow method, we identified four sample centers, subsequently assigning samples to the nearest cluster and updating cluster center points. Those process were repeated until cluster center points stabilized or a predetermined number of iterations was reached.

### 2.3 Definitions of Outcomes

The CVD outcome in UK Biobank including coronary heart disease (CHD), stroke, atrial fibrillation (AF), and heart failure (HF) was defined according to a previously reported study [31]. The outcomes were defined as International Classification of Diseases (ICD) edition 9 codes and edition 10 codes according to a previously reported study [31]. Person‐time of follow‐up was calculated from the date of the first repeat visit through the date of diagnosis, death, or withdrawal from the study, or end of the most recent follow‐up (November 2, 2022), whichever came first. In CHARLS, the primary outcome was incident CVD (stroke or cardiac events) from Wave 2 to Wave 5. The incidents of stroke and cardiac events were defined based on a self‐reported physician's diagnosis (“Has a doctor ever told you that you had any heart disease [myocardial infarction, coronary heart disease, angina, congestive heart failure, or other heart problems] or stroke?”), following previously reported studies in CHARLS [32].

### 2.4 Covariates

The following potential confounders were selected in two cohorts for analysis: age, sex (male or female), region (urban or rural), education level (below or higher education in UK Biobank; illiteracy, primary school, middle school, or high school or above in CHARLS), smoking status (never, previous smoker, or current smoker in UK Biobank; no or yes in CHARLS), drinking status (never, less than a month, or least once a month in UK Biobank; no or yes in CHARLS), dyslipidemia, systolic blood pressure, history of CKD, physical activity, socioeconomic status (self‐reported household income, occupation, education, and health insurance in CHARLS, Townsend deprivation index in UK biobank) and medications (antihypertensive, anti‐dyslipidemic, and antidiabetic).

### 2.5 Statistical Analysis

By utilizing the “cluster” and “factoextra” packages to perform K‐means cluster analysis on two assessments of TyG‐BRI, we identified five sample centers, subsequently assigning samples to the nearest cluster and updating cluster center points: “Class 1 (18.12, 19.72 for UK Biobank; 24.42, 24.90 for CHARLS),” “Class 2 (28.34, 30.39 for UK Biobank; 32.64, 41.45 for CHARLS),” “Class 3 (38.39, 44.46 for UK Biobank; 38.28, 33.63 for CHARLS),” “Class 4 (45.11, 50.46 for UK Biobank; 47.78, 49.21 for CHARLS),” “Class 5 (60.36, 61.56 for UK Biobank; 61.99, 64.29 for CHARLS)” (Figure 1).

**FIGURE 1:**
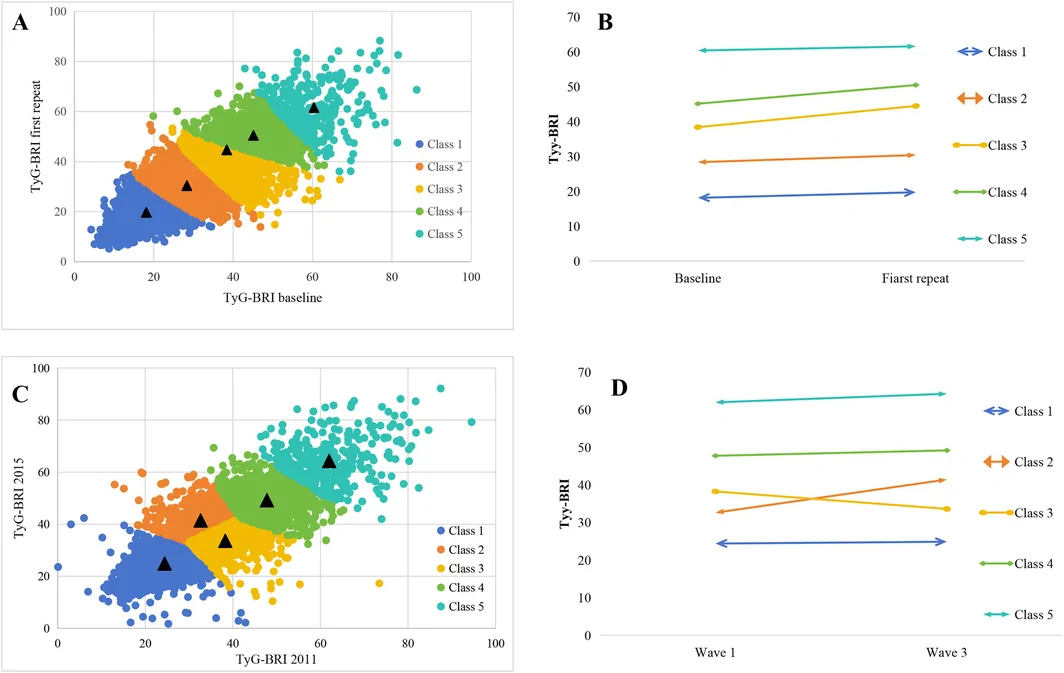
K‐means clustering in UK Biobank and CHARLS. (A) Clustering diagrams for TyG‐BRI _Baseline and TyG‐BRI _2012 in first repeat. (B) The TyG‐BRI clustering by K‐means clustering in UK Biobank. (C) Clustering diagrams for TyG‐BRI_2011 and TyG‐BRI _2015 in CHARLS. (D) The TyG‐BRI clustering by K‐means clustering in CHARLS.

Baseline characteristics were summarized by baseline (four subgroups according to TyG‐BRI quartiles), clustering (five class subgroups), and data were reported as percentages for categorical variables and as means ± standard deviations for continuous variables, and were compared by one‐way ANOVA or Kruskal–Wallis test for continuous variables and *χ*2 test for categorical variables.

We fitted three Cox‐proportional hazard models to investigate the longitudinal associations of baseline TyG‐BRI and longitudinal change of TyG‐BRI with the risk of incident CVD. Model 1 was adjusted for age and sex. Model 2 was adjusted for region, education level, smoking status, drinking status based on Model 1. Model 3 was further adjusted for dyslipidemia and medications (antihypertensive, anti‐dyslipidemic, and antidiabetic) based on Model 2. The proportional hazards assumptions were examined based on Schöenfeld residuals for all models. Restricted cubic splines (RCS) were used to assess whether there was a nonlinear association between baseline TyG‐BRI and CVD with 4 knots (at the 5th, 35th, 65th, and 95th percentiles). Kaplan–Meier cumulative incidence plots were generated to assess the associations of the change of TyG‐BRI with incident CVD during follow‐up, and the log‐rank test was used for statistical assessment. In subgroup analysis, the effects of sex, age, smoking, drinking and BMI on the associations of baseline and changes of TyG‐BRI with risk of CVD were further considered. The multiplicative interaction terms were fitted to examine the potential effects of subgroups. The multiple imputation with the Markov chain Monte Carlo method was performed to assign any missing covariate data. Two tailed *p* < 0.05 was considered statistical significance. All statistical analyses and figure preparation were conducted using SAS statistical software (version 9.4, Cary, NC) and R software (version 4.2.2, http://www.R‐project.org, The R Foundation).

## 3 Results

### 3.1 Baseline Characteristics

A total of 418,510 and 7923 individuals from the UK Biobank and CHARLS were included in Analysis 1. Baseline characteristics of the participants across the four subgroups in UK Biobank and CHARLS are provided in Supplementary Tables 1 and 2. In Analysis 2, a total of 12,971 and 4974 participants were included. Among the included individuals of UK Biobank, 6570 (50.65%) were women, and the mean (SD) age at baseline was 57.21 (7.40) years, and the prevalence of Class 1 to Class 5 was 39.25% (5091), 35.21% (4567), 16.25% (2108), 7.12% (923) and 2.17% (282). Among the CHA RLS, 2634 (52.96%) were women, and the mean (SD) age at baseline was 58.20 (8.68) years, and the prevalence of Class 1 to 5 was 33.79% (1567), 18.12% (840), 18.63% (864), 21.45% (995), and 8.00% (371). Baseline characteristics of the participants across the five subgroups in UK Biobank and CHARLS were provided in Supplementary Tables 3 and 4. Supplementary Tables 5 and 6 showed the baseline characteristics between the included and excluded in UK Biobank and CHARLS of Analysis 2.

### 3.2 Association of Baseline TyG‐BRI With Incident CVD

In Analysis 1, 41,012 CVD (9494 CHD, 11,428 stroke, 20,423 AF, and 11,621 HF) incident events in UK Biobank and 785 CVD (319 stroke and 536 cardiac events) in CHARLS were recorded. Table 1 showed the associations between TyG‐BRI with incident CVD. After adjusting for age, sex and other covariates, the HRs (95% CIs) from the Q2 to Q4 of TyG‐BRI were 1.08 (1.04–1.12), 1.12 (1.07–1.17), and 1.45 (1.39–1.52) in UK Biobank, respectively, compared with the lowest quartile of TyG‐BRI (Q1). Trend tests indicated a significant increase in CVD risk with increasing TyG‐BRI levels (*p* < 0.05). Multivariable‐adjusted RCS analyses presented positive linear associations between e TyG‐BRI with CVD risk (*p* for linearity < 0.001) (Figure 2). In CHARLS, individuals in Q2–Q4 of TyG‐BRI were associated with 23%, 44%, and 82% increased risk of CVD, comparison to those in the Q1, with the corresponding HRs were 1.12 (0.96–1.30), 1.21 (1.04–1.41) and 1.35 (1.13–1.61), respectively (Table 1). Trend test also showed a significant increase in CVD risk with increasing TyG‐BRI (*p* < 0.001), and there was also positive linear association between TyG‐BRI with CVD risk (Table 1; Figure 2). In the subgroup analysis, significant associations between the highest level of TyG‐BRI with CVD risk were observed in all subgroups in UK Biobank and CHARLS cohort (Supplemental Table 7). There were significant associations between highest level of TyG‐BRI with risk of CHD, stroke, AF and HF in UK Biobank, and with risk of incident stroke and cardiac events in CHARLS (Supplemental Figure 3; Supplemental Figure 4; Supplemental Table 8).

**TABLE 1:** Association of baseline TyG‐BRI with new‐onset CVD in UK Biobank and CHARLS.

Variable	Case, *n* (%)	Model 1^a^		Model 2^b^		Model 3^c^	
		HR (95CI)	*p*	HR (95CI)	*p*	HR (95CI)	*p*
UK Biobank, *N* = 418,510							
Quartiles							
Q 1 (< 20.88)	5628 (5.38)	1.00 (Ref)		1.00 (Ref)		1.00 (Ref)	
Q 2 (20.88–28.03)	8699 (8.32)	1.17 (1.13–1.21)	< 0.001	1.15 (1.11–1.20)	< 0.001	1.08 (1.04–1.12)	< 0.001
Q 3 (28.03–36.45)	11,089 (10.59)	1.33 (1.28–1.38)	< 0.001	1.28 (1.24–1.33)	< 0.001	1.12 (1.07–1.17)	< 0.001
Q 4 (≥ 36.45)	15,596 (14.90)	1.93 (1.86–1.99)	< 0.001	1.78 (1.72–1.82)	< 0.001	1.45 (1.39–1.52)	< 0.001
*p* for trend			< 0.001		< 0.001		< 0.001
CHARLS, *N* = 7923							
Quartiles							
Q 1 (< 26.85)	336 (16.94)	1.00 (Ref)		1.00 (Ref)		1.00 (Ref)	
Q 2 (26.85–34.58)	403 (20.36)	1.24 (1.06–1.46)	< 0.001	1.23 (1.06–1.22)	0.005	1.12 (0.96–1.30)	0.141
Q 3 (34.58–44.46)	478 (24.13)	1.52 (1.30–1.79)	< 0.001	1.45 (1.26–1.67)	< 0.001	1.21 (1.04–1.41)	0.015
Q 4 (≥ 44.46)	599 (30.25)	1.99 (1.70–2.34)	< 0.001	1.84 (1.59–2.11)	< 0.001	1.35 (1.13–1.61)	0.001
*p* for trend			< 0.001		< 0.001		< 0.001

^a^ Model 1 adjusted for age and sex.

^b^ Model 2 further adjusted for region, education level, smoking status, drinking status based on Model 1.

^c^ Model 3 further adjusted for dyslipidemia, systolic blood pressure, history of CKD, physical activity, socioeconomic status and medications (antihypertensive, anti‐dyslipidemic, and antidiabetic) based on Model 2.

**FIGURE 2:**
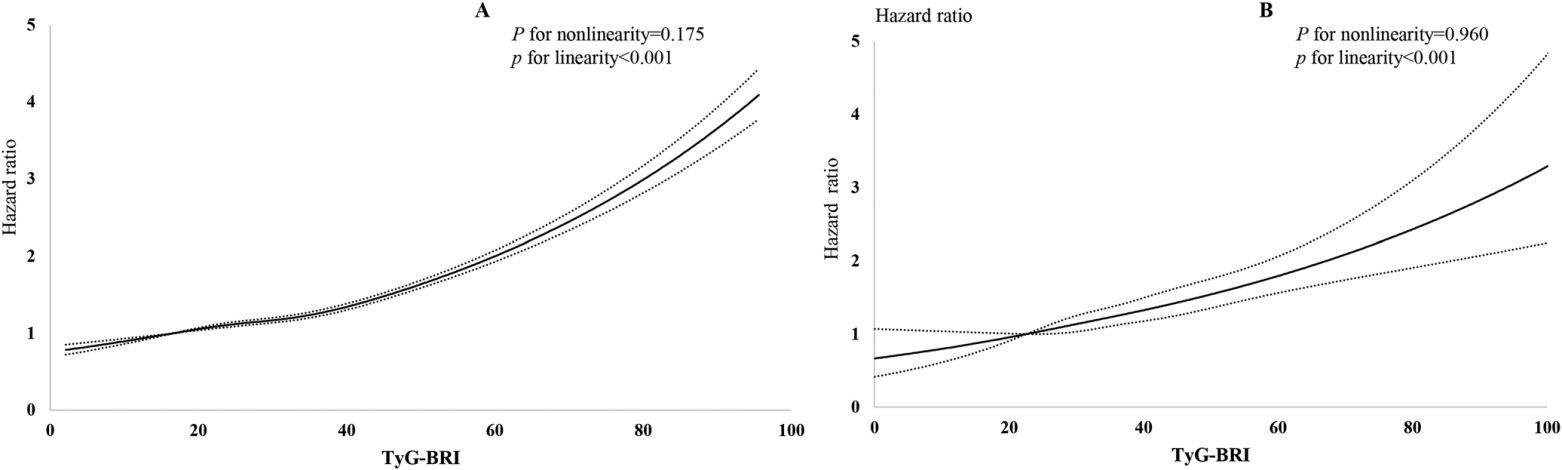
Association of baseline TyG‐BRI with risk of CVD (A: UK Biobank; B: CHARLS). Hazard ratios and 95% CIs derived from restricted cubic spline regression, with knots placed at the 5th, 35th, 65th, and 95th percentiles of the distribution of baseline TyG‐BRI. The reference point for TyG‐BRI is the midpoint (16.75 and 22.65 for UK Biobank and CHARLS) of the reference group from categorical analysis. The lowest 1% and highest 1% of participants were not shown in the figures for small sample sizes. Hazard ratios were adjusted for the same variables as the multivariable‐adjusted model in Table 1.

### 3.3 Association of Changes in TyG‐BRI With Incident CVD

The median follow‐up periods were 9.7 years in the UK Biobank and 5.0 years in the CHARLS. During the follow‐up, 1154 CVD (242 CHD, 273 stroke, 678 AF and 273 HF) incident events in UK Biobank and 550 CVD (319 stroke and 536 cardiac events) in CHARLS were recorded. Participants in Class 3 to 5 had higher cumulative incidence of CVD in both UK Biobank and CHARLS (log‐rank *p* < 0.001; Figure 3). In comparison with Class 1, the risk of CVD in Class 3 was significantly higher (HR = 1.23, 95% 1.02–1.49), as was the risk in Class 4 (HR = 1.48, 95% 1.17–1.87) and in Class 5 (HR = 2.58, 95% 1.86–3.58) of UK Biobank (Table 2). However, there was no significant association between Class 2 with risk of CVD. In CHARLS, individuals in Class 2, Class 3, Class 4, and Class 5 were associated with 23%, 42%, 45%, and 63% increased risk of CVD, compared to those in Class 1, with the corresponding HR were 1.23 (1.02–1.55), 1.42 (1.11–1.83), 1.45 (1.16–1.89), and 1.63 (1.16–2.28), respectively (Table 2). In the subgroup analysis, significant associations between Class 5 with CVD risk were observed in all subgroups in UK Biobank and CHARLS cohort (Supplemental Table 9). In UK Biobank, there were significant associations between Class 5 with risk of CHD, AF and HF (Supplemental Figure 5; Supplementary Table 8). Similarly, participants in Class 3, Class 4, and Class 5 presented significantly increased risks of incident stroke and cardiac events than those in Class 1 in CHARLS (Supplemental Figure 6; Supplementary Table 10).

**FIGURE 3:**
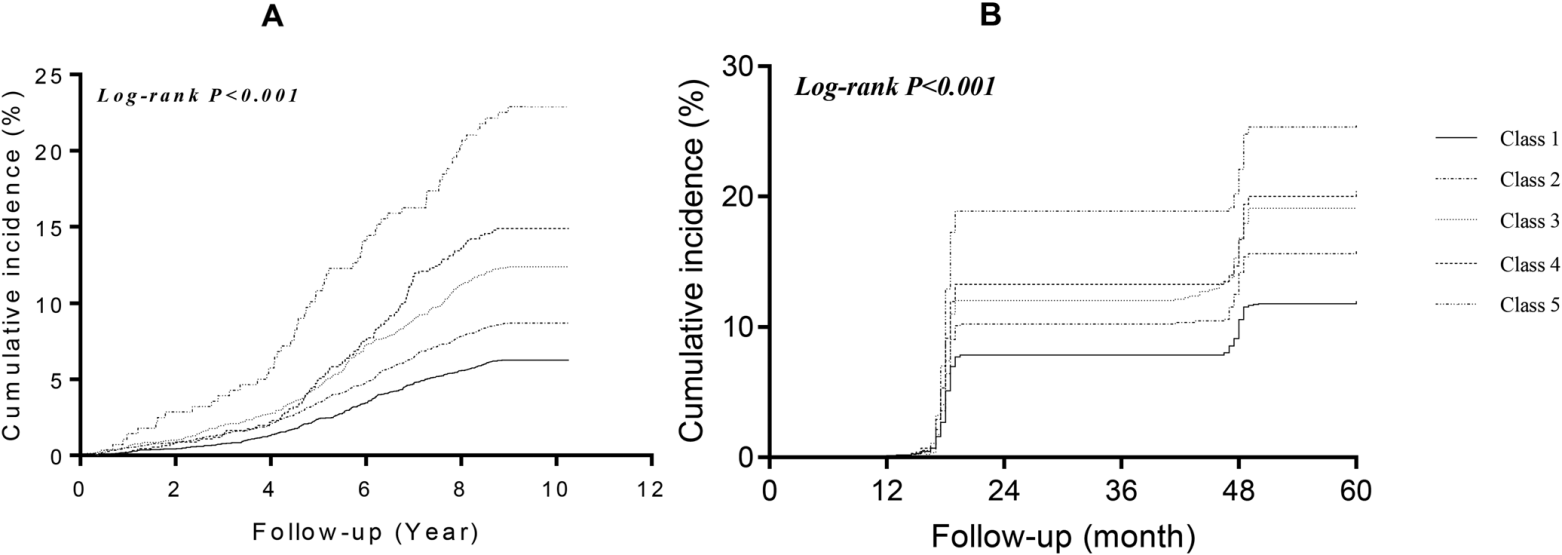
Kaplan–Meier curves for the cumulative risk of CVD according to different TyG‐BRI classes in UK Biobank and CHARLS (A: UK Biobank; B: CHARLS).

**TABLE 2:** Association of different TyG‐BRI classes with new‐onset CVD in UK Biobank and CHARLS.

Variable	Case, *n* (%)	Model 1^a^		Model 2^b^		Model 3^c^	
		HR (95CI)	*p*	HR (95CI)	*p*	HR (95CI)	*p*
UK Biobank, *N* = 11,682							
Categories							
Class 1	313 (6.15)	1.00 (Ref)		1.00 (Ref)		1.00 (Ref)	
Class 2	389 (8.52)	1.04 (0.89–1.23)	0.605	1.20 (0.87–1.20)	0.810	0.96 (0.82–1.12)	0.654
Class 3	255 (12.10)	1.44 (1.20–1.72)	< 0.001	1.37 (1.14–1.65)	< 0.001	1.23 (1.02–1.49)	0.027
Class 4	134 (14.52)	1.82 (1.45–2.27)	< 0.001	1.70 (1.36–2.14)	< 0.001	1.48 (1.17–1.87)	0.001
Class 5	63 (22.34)	3.49 (2.55–4.79)	< 0.001	3.18 (2.31–4.38)	< 0.001	2.58 (1.86–3.58)	< 0.001
*p* for trend			< 0.001		< 0.001		< 0.001
CHARLS, *N* = 4637							
Categories							
Class 1	188 (12.00)	1.00 (Ref)		1.00 (Ref)		1.00 (Ref)	
Class 2	134 (15.95)	1.34 (1.07–1.68)	0.010	1.35 (1.08–1.70)	0.009	1.23 (1.02–1.55)	0.047
Class 3	165 (19.10)	1.60 (1.30–1.98)	< 0.001	1.62 (1.31–2.00)	< 0.001	1.42 (1.11–1.83)	0.006
Class 4	203 (20.40)	1.71 (1.39–2.10)	< 0.001	1.74 (1.41–2.14)	< 0.001	1.45 (1.16–1.89)	0.001
Class 5	95 (25.61)	2.16 (1.66–2.80)	< 0.001	2.20 (1.69–2.85)	< 0.001	1.63 (1.16–2.28)	0.003
*p* for trend			< 0.001		< 0.001		< 0.001

^a^ Model 1 adjusted for age and sex.

^b^ Model 2 further adjusted for region, education level, smoking status, drinking status based on Model 1.

^c^ Model 3 further adjusted for dyslipidemia, systolic blood pressure, history of CKD, physical activity, socioeconomic status, and medications (antihypertensive, anti‐dyslipidemic, and antidiabetic) based on Model 2.

## 4 Discussion

Based on two prospective cohort datasets, the present study explored the associations of baseline TyG‐BRI levels and longitudinal TyG‐BRI changes with incident CVD risk. Our results demonstrated that CVD incidence rose progressively across ascending quartiles of baseline TyG‐BRI, with a positive linear dose–response relationship observed between baseline TyG‐BRI and CVD risk. Furthermore, participants with persistently elevated TyG‐BRI levels (Classes 3–5) during follow‐up exhibited a significantly higher risk of developing CVD. Collectively, these findings confirm that both higher baseline TyG‐BRI and longitudinal increases in TyG‐BRI are independently correlated with elevated CVD risk. This evidence highlights that monitoring baseline status and long‐term dynamic changes in TyG‐BRI may facilitate early screening and identification of individuals at high risk of CVD.

In recent years, the TyG index has been widely recognized as a reliable surrogate marker for IR due to its simple calculation procedure and favorable sensitivity and specificity for IR identification. Accumulating evidence from cross‐sectional studies, prospective cohorts, and meta‐analyses has confirmed that elevated TyG index serves as an independent risk factor for CVD [12, 33]. Furthermore, combining the TyG index with adiposity indicators yields superior predictive performance for CVD risk compared with the standalone TyG index [29]. Unlike conventional anthropometric parameters including BMI, WC, and hip circumference, BRI can comprehensively characterize body shape and more accurately reflect overall body fat distribution and visceral fat accumulation [19]. Although the predictive value of composite TyG indices (TyG‐BMI, TyG‐WC, and TyG‐WHtR) for CVD risk has been well documented [17, 18], only a limited number of studies have explored the associations between TyG‐BRI and disease incidence [21, 34]. Consistent with prior investigations, the present study demonstrated that higher baseline TyG‐BRI was independently correlated with increased CVD risk in both the UK Biobank and CHARLS cohorts, and a positive linear dose–response relationship was observed between TyG‐BRI and CVD risk. Taken together, these findings suggest that TyG‐BRI, as a novel biomarker integrating IR status and body shape characteristics, possesses promising predictive potential for CVD risk evaluation.

TG, FBG, WC and height, the core parameters for calculating TyG and BRI—are well‐recognized time‐varying exposures. Given the dynamic temporal variations in TyG and BRI levels, multiple studies have explored the association of longitudinal changes in these two indices with CVD events [35, 36]. For instance, Huo et al. revealed that persistently elevated TyG‐BMI was independently associated with an increased stroke risk among middle‐aged and older adults [24]. Similarly, Ren et al. classified participants into distinct subgroups via K‐means cluster analysis based on longitudinal TyG‐WHtR trajectories and found that individuals with moderately elevated and progressively increasing TyG‐WHtR levels had a higher risk of incident CVD [25]. Distinct from previous studies, the present study adopted cluster analysis to categorize longitudinal TyG‐BRI trajectories in both the UK Biobank and CHARLS cohorts. Each clustered subgroup represented a unique phenotypic population. Notably, participants with persistently low TyG‐BRI exhibited the lowest CVD risk, whereas those with sustained high TyG‐BRI had the highest CVD susceptibility.

To our knowledge, this is the first study to systematically evaluate the predictive value of both baseline levels and longitudinal trajectories of TyG‐BRI for CVD risk across European and Asian populations. By focusing on dynamic alterations in TyG‐BRI during follow‐up, our findings provide novel evidence supporting the independent association between TyG‐BRI and CVD risk. This study enriches the current evidence base for CVD prevention and risk stratification, demonstrating that longitudinal changes in TyG‐BRI can serve as a clinically practical marker to identify individuals at elevated CVD risk. As TyG‐BRI is calculated based on routinely measured TyG (TG and FBG) and BRI (WC and height) parameters, it can be conveniently derived from a single simultaneous blood and anthropometric assessment. Accordingly, TyG‐BRI represents a cost‐effective and accessible surrogate alternative to the gold‐standard euglycemic–hyperinsulinemic clamp technique. Furthermore, given that TG, FBG, WC, and height are universally available routine indicators in primary healthcare settings, TyG‐BRI exhibits excellent generalizability and practicality for both clinical practice and large‐scale epidemiological research. Future large‐scale, multi‐ethnic studies are warranted to further validate and expand the clinical application of TyG‐BRI.

Our findings were derived from two large, methodologically rigorous prospective cohorts covering different geographical regions and ethnic populations with long‐term follow‐up. The consistent results observed across the two cohorts strengthen the generalizability of our findings and support their extrapolation to broader populations. Nevertheless, several limitations of the present study should be acknowledged. First, potential selection bias may exist, as participants with missing TyG‐BRI data were excluded from the analysis. Notably, only a limited number of participants in the UK Biobank completed the first follow‐up assessment, which may further introduce selection bias. Second, repeated TyG‐BRI measurements were performed at irregular intervals in both the UK Biobank and CHARLS cohorts. Heterogeneous time intervals for repeated assessments between the two cohorts may affect the clinical interpretation of our results, particularly regarding the optimal monitoring frequency of TyG‐BRI. Third, as an observational study, our analysis cannot establish definitive causal relationships between TyG‐BRI dynamics and CVD risk. Although we adjusted for a comprehensive set of confounding factors, the volunteer‐based nature of the UK Biobank cohort may limit population representativeness, as participants tend to be healthier and socioeconomically advantaged compared with the general UK population.

In addition, the baseline ages of the two cohorts and the differences in risk factor exposure between the two cohort studies, as risk factors such as smoking and alcohol consumption may also contribute to the difference in CVD risk between these two cohorts. Fourth, dietary data are not collected in the CHARLS study; thus, we could not eliminate the potential confounding effect of dietary patterns on TyG levels. Fifth, CVD ascertainment in CHARLS relied on self‐reported physician‐diagnosed outcomes, which may introduce misclassification bias. However, self‐reported disease history has been validated and demonstrated acceptable reliability in previous epidemiological studies.

In conclusion, based on data from the UK Biobank and CHARLS cohorts, our prospective study demonstrated that higher baseline TyG‐BRI levels and persistently elevated TyG‐BRI trajectories during follow‐up are independently associated with an increased risk of incident CVD.

## Funding

This work was supported by Jiangsu Province Young Science and Technology Talent Support Program (JSTJ‐2025‐474).

## Ethics Statement

UK Biobank was constructed under ethical approval obtained by the North West Multi‐Centre Research Ethics Committee (REC reference: 11/NW/03820). The current analyses were carried out under Application Number 91185.

## Conflicts of Interest

The authors declare no conflicts of interest.

## Supporting information


**Supplementary Table 1.** Characteristics of the study participants according to the baseline TyG‐BRI in UK Biobank (N = 418,510).
**Supplemental Table 2**. Baseline characteristics of the study participants according to the quartiles of baseline TyG‐BRI (Analysis 1, *N* = 7923).
**Supplementary Table 3**. Characteristics of the study participants according to the different TyG‐BRI classes in UK Biobank (*N* = 11,682).
**Supplemental Table 4**. Baseline characteristics of the study participants according to the change in the TyG‐BRI (Analysis 2, *N* = 4637).
**Supplemental Table 5**. Subgroup analysis of HRs (95% CI) of baseline TyG‐BRI with new‐onset CVD in CHARLS and UK Biobank.
**Supplemental Table 6**. Association of baseline TyG‐BRI with new‐onset CVD in UK Biobank and CHARLS.
**Supplemental Table 7**. Subgroup analysis of HRs (95% CI) of TyG‐BRI classes with new‐onset CVD in CHARLS and UK Biobank.
**Supplemental Table 8**. Association of different TyG‐BRI classes with new‐onset CVD in UK Biobank and CHARLS.
**Supplemental Figure 1**. Flowchart of sample selection and the exclusion criteria in UK Biobank.
**Supplemental Figure 2**. Flowchart of sample selection and the exclusion criteria in CHARLS.
**Supplemental Figure 3**. Association of baseline TyG‐BRI with risk of CHD, stroke, AF and HF in UK Biobank. Hazard ratios and 95% CIs derived from restricted cubic spline regression, with knots placed at the 5th, 35th, 65th, and 95th percentiles of the distribution of baseline TyG‐BRI. The reference point for TyG‐BRI is the midpoint (16.75) of the reference group from categorical analysis. The lowest 1% and highest 1% of participants were not shown in the figures for small sample sizes. Hazard ratios were adjusted for the same variables as multivariable‐adjusted model in Table 1.
**Supplemental Figure 4**. Association of baseline TyG‐BRI with risk of stroke and cardiac events in CHARLS. Hazard ratios and 95% CIs derived from restricted cubic spline regression, with knots placed at the 5th, 35th, 65th, and 95th percentiles of the distribution of baseline TyG‐BRI. The reference point for TyG‐BRI is the midpoint (22.65) of the reference group from categorical analysis. The lowest 1% and highest 1% of participants were not shown in the figures for small sample sizes. Hazard ratios were adjusted for the same variables as multivariable‐adjusted model in Table 1.
**Supplemental Figure 5**. Kaplan–Meier curves for the cumulative risk of CVD risk according to different TyG‐BRI classes in UK Biobank (A: CHD; B: stroke; C: AF; D: HF).
**Supplemental Figure 6**. Kaplan–Meier curves for the cumulative risk of CVD risk according to different TyG‐BRI classes in CHARLS (A: stroke; B: cardiac events).

**Supplemental Table 9.** Subgroup analysis of HRs (95% CI) of TyG‐BRI classes with new‐onset CVD in CHARLS and UK Biobank.
**Supplemental Table 10**. Association of different TyG‐BRI classes with new‐onset CVD in UK Biobank and CHARLS.

## Data Availability

The dataset supporting the conclusions of this article is available in the public domain UK Biobank Resource (www.ukbiobank.ac.uk/).
