# Supplementary material for: Associations of the Baseline Level and Change in Triglyceride Glucose‐Body Roundness Index With Cardiovascular Diseases: Two Large Prospective Cohorts in Europe and Asia
Source: Kaohsiung J Med Sci. 2026 Oct 22:e70305. Online ahead of print. doi: 10.1002/kjm2.70305 (PMC13688557; doi:10.1002/kjm2.70305)
Supplement: Supplementary file 1 [file KJM2-9999-e70305-s001.docx]

**Supplementary Table S1.** Characteristics of the study participants according to the baseline TyG-BRI in UK Biobank (N=418,510)

| Characteristics | Q 1 (<20.88) | Q 2 (20.88-28.03) | Q 3 (28.03-36.45) | Q 4 (≥36.45) | *P* trend |
| --- | --- | --- | --- | --- | --- |
| No. of subjects | 104,606 | 104,574 | 104.667 | 104,663 |  |
| Age, years | 54.220±8.19 | 56.31±8.10 | 57.38±7.92 | 58.01±7.66 | <0.001 |
| Sex, n (%) |  |  |  |  |  |
| Male | 80,576(77.03) | 55,497(53.05) | 44,553(42.57) | 46,759(44.68) | <0.001 |
| Female | 24,030(22.97) | 49,095(46.95) | 60,114(57.43) | 57,904(55.32) |  |
| Living place, n (%) |  |  |  |  |  |
| Urban | 79,832(77.18) | 81,198(78.46) | 82,521(79.64) | 85,320(82.25) | <0.001 |
| Rural | 24,774(22.82) | 23,376(21.54) | 22,146(20.36) | 19,343(17.75) |  |
| White ethnicity (n, %) | 99,863(95.47) | 98,724(94.41) | 98,050(93.68) | 97,506(93.16) | <0.001 |
| Higher education (n, %) | 48,429(46.30) | 41,723(39.90) | 26,979(35.33) | 30.882(29.51) | <0.001 |
| BMI, kg/m^2^ | 22.87±2.20 | 25.67±2.17 | 28.04±2.46 | 32.65±4.09 | <0.001 |
| Smoking (n, %) |  |  |  | |  |
| Never | 64,769(61.92) | 59,563(56.96) | 54,765(52.32) | 50,128(47.89) | <0.001 |
| Previous smoker | 29,726(28.42) | 34,096(32.60) | 38,627(36.90) | 43,045(41.13) |  |
| Current smoker | 10,111(9.67) | 10,915(10.44) | 11,275(10.77) | 11,490(10.98) |  |
| Alcohol intake (n, %) |  |  |  | |  |
| Never | 3,871(3.80) | 3,953(3.78) | 4,356(4.16) | 5,919(3.60) | <0.001 |
| Less than a month | 3,404(3.25) | 3,277(3.13) | 3,703(3.54) | 5230(5.00) |  |
| Least once a month | 97,231(92.05) | 97,345(93.09) | 96,608(92.30) | 93,514(89.35) |  |
| Blood pressure |  |  |  | |  |
| SBP, mm Hg | 130.64±18.21 | 137.11±18.11 | 140.82±17.86 | 143.18±17.72 | <0.001 |
| DBP, mm Hg | 77.91±9.52 | 81.59±9.62 | 84.06±9.68 | 85.81±9.80 | <0.001 |
| Diabetes mellitus (n, %) | 1,176(1.12) | 2,272(2.17) | 4,529(4.33) | 12,946(12.37) | <0.001 |
| Hyperlipidemia (%) | 8,515(8.14) | 16,451(15.73) | 24,197(23.12) | 34,630(33.09) | <0.001 |
| Medications (n, %) |  |  |  |  | <0.001 |
| Cholesterol-lowering medication | 5.351(5.12) | 8,713(8.33) | 11,639(11.12) | 16,646(15.90) |  |
| Blood pressure medication | 5,561(5.32) | 3,436(3.29) | 2,328(2.22) | 1,659(1.59) |  |
| Insulin | 2,889(2.76) | 1,222(1.17) | 670(0.64) | 500(0.48) |  |
| Townsend Deprivation Index | -1.55±2.95 | -1.54±2.96 | -1.37±3.05 | -0.84±3.25 | <0.001 |

BMI: body mass index; SBP: systolic blood pressure; DBP: diastolic blood pressure;

Continuous variables are expressed as mean± standard deviation, or as median (interquartile range). Categorical variables are expressed as frequency (percent).

**Supplemental Table 2.** Baseline characteristics of the study participants according to the quartiles of baseline TyG-BRI (Analysis 1, N=7,923).

| Variable | Quartiles of TyG-BRI (N=7,923) | | | |  |
| --- | --- | --- | --- | --- | --- |
|  | Q 1(<26.85) | Q 2(26.85-34.58) | Q 3(34.58-44.46) | Q 4(≥44.46) |  |
| No. of subjects | 1,983 | 1,979 | 1,981 | 1,980 |  |
| Age, years | 58.89±9.41 | 58.45±9.25 | 58.56±9.40 | 59.73±9.45 | <0.001 |
| Sex, n (%) |  |  |  |  |  |
| Male | 1,353(68.23) | 1,051(53.11) | 835(42.15) | 522(26.36) | <0.001 |
| Female | 630(31.77) | 928(46.89) | 1,146(57.85) | 1,458(73.64) |  |
| Living place, n (%) |  |  |  |  |  |
| Urban | 511(25.77) | 616(31.13) | 736(37.15) | 801(40.45) | <0.001 |
| Rural | 1,472(74.23) | 1,363(68.87) | 1,245(62.85) | 1,179(59.85) |  |
| Education level, n (%) |  |  |  |  |  |
| Illiteracy | 520(26.22) | 551(27.84) | 592(29.88) | 719(36.31) | 0.052 |
| Primary school | 877(44.23) | 818(41.33) | 787(39.73) | 748(37.78) |  |
| Middle school | 400(20.17) | 400(20.21) | 389(19.64) | 362(18.28) |  |
| High school or above | 186(9.38) | 210(10.61) | 213(10.75) | 151(7.63) |  |
| Medical history |  |  |  |  |  |
| Dyslipidemia, n (%) | 60(3.03) | 99(5.00) | 149(7.52) | 285(14.39) | <0.001 |
| Diabetes mellitus, n (%) | 40(2.02) | 60(3.03) | 127(6.41) | 202(10.20) | <0.001 |
| Hypertension, n (%) | 394(19.87) | 413(20.87) | 44(22.41) | 450(22.73) | 0.014 |
| Smoking, n (%) |  |  |  |  |  |
| No | 848(42.76) | 1,113(56.24) | 1,295(65.37) | 1,524(76.97) | <0.001 |
| Yes | 1,135(57.42) | 866(43.76) | 686(34.63) | 456(23.03) |  |
| Drinking, n (%) |  |  |  |  |  |
| No | 999(50.38) | 1,133(57.25) | 1,221(61.64) | 1,409(71.16) | <0.001 |
| Yes | 984(49.62) | 846(42.75) | 760(38.36) | 571(28.84) |  |
| BMI (kg/m^2^) | 19.98(18.67-21.32) | 22.16(20.73-23.47) | 24.16(22.68-25.65) | 26.92(24.92-28.90) | <0.001 |
| SBP, mmHg | 124.74±20.01 | 127.01±19.24 | 131.43±21.43 | 136.01±22.05 | <0.001 |
| DBP, mmHg | 73.22±11.25 | 74.65±11.91 | 76.33±11.51 | 78.21±11.45 | <0.001 |

FBG: Fasting blood glucose; BMI: body mass index; SBP: systolic blood pressure; DBP: diastolic blood pressure;

Continuous variables are expressed as mean± standard deviation, or as median (interquartile range). Categorical variables are expressed as frequency (percent).

**Supplementary Table S3.** Characteristics of the study participants according to the different TyG-BRI classes in UK Biobank (N=11,682)

| Characteristics | Class 1 | Class 2 | Class 3 | Class 4 | Class 5 | *P* trend |
| --- | --- | --- | --- | --- | --- | --- |
| No. of subjects | 5,091 | 4,567 | 2,108 | 923 | 282 |  |
| Age, years | 55.95±7.60 | 57.70±7.23 | 58.48±6.96 | 58.55±7.29 | 58.15±6.59 | <0.001 |
| Sex, n (%) |  |  |  |  |  |  |
| Male | 3,341(65.63) | 1,791(39.22) | 753(35.72) | 390(42.25) | 126(44.68) | <0.001 |
| Female | 1,750(34.37) | 2,776(60.78) | 1,355(64.28) | 533(57.75) | 156(55.32) |  |
| Living place, n (%) |  |  |  |  |  |  |
| Urban | 4,278(77.18) | 3,889(85.87) | 1,814(86.46) | 828(90.10) | 255(91.73) | <0.001 |
| Rural | 813(22.82) | 678(14.13) | 294(13.54) | 95(9.9) | 27(8.27) |  |
| White ethnicity (n, %) | 4,975(97.72) | 4,444(97.31) | 2,047(97.11) | 901(97.62) | 276(97.87) | <0.001 |
| Higher education (n, %) | 2,758(54.17) | 2,48(47.03) | 920(43.64) | 367(39.76) | 101(35.82) | <0.001 |
| BMI, kg/m^2^ | 23.45±2.27 | 26.95±2.29 | 30.21±2.75 | 32.02±3.17 | 37.98±3.94 | <0.001 |
| Smoking (n, %) |  |  |  |  |  |  |
| Never | 3,397(66.73) | 2,611(57.17) | 1,096(51.99) | 464(50.27) | 135(47.87) | <0.001 |
| Previous smoker | 1,415(27.79) | 1,665(36.46) | 850(40.32) | 374(40.52) | 121(42.91) |  |
| Current smoker | 279(5.48) | 291(6.37) | 162(7.69) | 85(9.21) | 26(9.22) |  |
| Alcohol intake (n, %) |  |  |  |  |  |  |
| Never | 146(2.87) | 117(2.56) | 62(2.94) | 37(4.01) | 16(5.67) | <0.001 |
| Less than a month | 118(2.32) | 97(2.00) | 57(2.70) | 34(3.68) | 12(4.26) |  |
| Least once a month | 4,827(94.81) | 4,353(95.31) | 1,989(94.35) | 852(92.31) | 254(90.07) |  |
| Blood pressure |  |  |  |  |  |  |
| SBP, mm Hg | 133.29±17.98 | 139.66±17.17 | 142.30±17.14 | 142.53±17.38 | 142.71±17.14 | <0.001 |
| DBP, mm Hg | 78.57±9.35 | 82.83±9.54 | 85.01±9.15 | 85.39±9.50 | 85.70±8.59 | <0.001 |
| Diabetes mellitus (n, %) | 60(1.18) | 121(2.65) | 134(6.36) | 104(11.27) | 77(27.30) | <0.001 |
| Hyperlipidemia (%) | 882(17.32) | 1,465(32.08) | 938(44.50) | 470(50.92) | 182(64.54) | <0.001 |
| Medications (n, %) |  |  |  |  |  | <0.001 |
| Cholesterol-lowering medication | 23(0.45) | 25(0.55) | 11(0.52) | 3(0.33) | 5(1.77) |  |
| Blood pressure medication | 278(5.46) | 399(8.74) | 261(12.38) | 137(14.84) | 52(18.44) |  |
| Insulin | 255(5.01) | 124(2.72) | 37(1.76) | 19(2.06) | 4(1.42) |  |
| Townsend Deprivation Index | -2.25±2.61 | -2.12±2.62 | -1.87±2.76 | -1.52±2.91 | -0.98±3.05 | <0.001 |

BMI: body mass index; SBP: systolic blood pressure; DBP: diastolic blood pressure;

Continuous variables are expressed as mean± standard deviation, or as median (interquartile range). Categorical variables are expressed as frequency (percent).

**Supplemental Table 4.** Baseline characteristics of the study participants according to the change in the TyG-BRI (Analysis 2, N=4,637).

| Variable | Class 1 | Class 2 | Class 3 | Class 4 | Class 5 | *P* value |
| --- | --- | --- | --- | --- | --- | --- |
| No. of subjects | 1567 | 840 | 864 | 995 | 371 |  |
| Age, years | 58.19±8.70 | 57.17±8.35 | 58.69±8.60 | 58.07±8.77 | 59.00±8.58 | 0.126 |
| Sex, n (%) |  |  |  |  |  |  |
| Male | 1059(67.58) | 354(42.14) | 412(47.69) | 312(31.36) | 52(14.02) | <0.001 |
| Female | 508(32.42) | 486(57.86) | 452(52.31) | 683(68.64) | 319(85.98) |  |
| Living place, n (%) |  |  |  |  |  |  |
| Urban | 395(25.21) | 292(34.76) | 283(32.75) | 371(37.29) | 147(39.62) | <0.001 |
| Rural | 1172(74.69) | 548(65.24) | 581(67.25) | 624(62.71) | 224(60.38) |  |
| Education level, n (%) |  |  |  |  |  |  |
| Illiteracy | 395(25.21) | 225(26.79) | 247(28.59) | 312(31.36) | 152(40.97) | <0.001 |
| Primary school | 690(44.03) | 345(41.07) | 366(42.36) | 400(40.20) | 138(37.20) |  |
| Middle school | 334(21.31) | 184(21.90) | 172(19.91) | 200(20.10) | 65(17.52) |  |
| High school or above | 148(9.44) | 86(10.24) | 79(9.14) | 83(8.34) | 16(4.31) |  |
| Medical history |  |  |  |  |  |  |
| Dyslipidemia, n (%) | 48(3.06) | 53(6.31) | 60(6.94) | 121(12.16) | 58(15.63) | <0.001 |
| Diabetes mellitus, n (%) | 37(2.36) | 22(2.62) | 60(6.94) | 82(8.24) | 42(11.32) | <0.001 |
| Hypertension, n (%) | 332(21.19) | 175(20.83) | 211(24.42) | 221(22.21) | 95(25.61) | 0.065 |
| Smoking, n (%) |  |  |  |  |  |  |
| No | 693(44.22) | 549(65.36) | 545(63.08) | 746(74.97) | 317(85.44) | <0.001 |
| Yes | 874(55.78) | 291(34.64) | 319(36.92) | 249(25.03) | 54(14.56) |  |
| Drinking, n (%) |  |  |  |  |  |  |
| No | 793(50.61) | 525(62.50) | 528(61.11) | 686(68.94) | 294(79.25) | <0.001 |
| Yes | 774(49.39) | 315(37.50) | 336(38.89) | 309(31.06) | 77(20.75) |  |
| BMI (kg/m^2^) | 20.42  (19.12-21.85) | 23.10  (21.64-24.32) | 23.47  (22.07-24.92) | 26.04  (24.33-27.63) | 28.84  (27.13-30.88) | <0.001 |
| SBP, mmHg | 123.33±19.09 | 128.17±19.22 | 129.54±19.87 | 133.77±20.17 | 137.96±19.85 | <0.001 |
| DBP, mmHg | 72.75±11.00 | 76.45±12.14 | 75.58±11.61 | 77.55±11.58 | 79.04±10.49 | <0.001 |

FBG: Fasting blood glucose; BMI: body mass index; SBP: systolic blood pressure; DBP: diastolic blood pressure;

Continuous variables are expressed as mean± standard deviation, or as median (interquartile range). Categorical variables are expressed as frequency (percent).

**Supplemental Table 5.** Subgroup analysis of HRs (95% CI) of baseline TyG-BRI with new-onset CVD in CHARLS and UK Biobank.

| Characteristics | Q 1 | Q 2 | Q 3 | Q 4 | *P* value | *P-_interaction_* |
| --- | --- | --- | --- | --- | --- | --- |
| **UK Biobank, N=418,510** | | | | | | |
| Sex |  |  |  |  |  |  |
| Male | 1.00(Ref) | 1.12(1.06-1.18) | 1.20(1.13-1.28) | 1.59(1.49-1.69) | <0.001 | 0.016 |
| Female | 1.00(Ref) | 0.96(0.91-1.02) | 0.97(0.91-1.04) | 1.24(1.16-1.33) | <0.001 |  |
| Age, years |  |  |  |  |  |  |
| <60 | 1.00(Ref) | 1.18(1.10-1.26) | 1.36(1.26-1.47) | 1.78(1.65-1.92) | <0.001 | <0.001 |
| ≥60 | 1.00(Ref) | 0.99(0.94-1.05) | 1.00(0.96-1.05) | 1.28(1.21-1.35) | <0.001 |  |
| Smoking |  |  |  |  |  |  |
| No | 1.00(Ref) | 1.17(1.10-1.25) | 1.23(1.15-1.30) | 1.63(1.54-1.73) | <0.001 | 0.169 |
| Yes | 1.00(Ref) | 1.08(1.03-1.14) | 1.17(1.11-1.23) | 1.54(1.47-1.61) | <0.001 |  |
| Drinking |  |  |  |  |  |  |
| No | 1.00(Ref) | 0.95(0.80-1.12) | 1.02(0.87-1.19) | 1.41(1.22-1.63) | <0.001 | 0.025 |
| Yes | 1.00(Ref) | 1.03(0.86-1.22) | 1.19(1.018-1.40) | 1.62(1.39-1.89) | <0.001 |  |
| BMI, kg/m^2^ |  |  |  |  |  |  |
| <25 | 1.00(Ref) | 1.02(0.98-1.08) | 1.06(0.98-1.14) | 1.21(0.95-1.14) | 0.063 | <0.001 |
| ≥25 | 1.00(Ref) | 1.12(1.04-1.21) | 1.17(1.08-1.26) | 1.52(1.41-1.63) | <0.001 |  |
| **CHARLS, N=7,923** | | | | | | |
| Sex |  |  |  |  |  |  |
| Male | 1.00(Ref) | 1.13(0.93-1.38) | 1.20(0.96-1.50) | 1.52(1.15-2.01) | <0.001 | <0.001 |
| Female | 1.00(Ref) | 1.09(0.86-1.37) | 1.18(0.94-1.48) | 1.44(1.08-1.59) | <0.001 |  |
| Age, years |  |  |  |  |  |  |
| <60 | 1.00(Ref) | 1.10(0.89-1.35) | 1.24(1.01-1.54) | 1.41(1.10-1.82) | 0.004 | <0.001 |
| ≥60 | 1.00(Ref) | 1.13(0.97-1.31) | 1.31(1.13-1.52) | 1.61(1.39-1.87) | <0.001 |  |
| Smoking |  |  |  |  |  |  |
| No | 1.00(Ref) | 1.24(1.01-1.55) | 1.29(1.04-1.60) | 1.39(1.10-1.76) | 0.010 | <0.001 |
| Yes | 1.00(Ref) | 1.03(0.84-1.28) | 1.17(0.93-1.48) | 1.40(1.04-1.89) | 0.027 |  |
| Drinking |  |  |  |  |  |  |
| No | 1.00(Ref) | 1.09(0.89-1.33) | 1.20(0.98-1.47) | 1.29(1.03-1.62) | 0.018 | 0.548 |
| Yes | 1.00(Ref) | 1.18(0.94-1.46) | 1.22(0.96-1.55) | 1.45(1.08-1.95) | 0.018 |  |
| BMI, kg/m^2^ |  |  |  |  |  |  |
| <24 | 1.00(Ref) | 1.17(1.04-1.31) | 1.38(1.23-1.54) | 1.73(1.54-1.93) | <0.001 | <0.001 |
| ≥24 | 1.00(Ref) | 1.50(0.80-2.81) | 1.92(1.09-3.39) | 2.18(1.25-3.80) | <0.001 |  |

In the multivariate models, confounding factors such as age, sex, region, education level, smoking status, drinking status, dyslipidemia, systolic blood pressure, history of CKD, physical activity, socioeconomic status and medications (anti-hypertensive, anti-dyslipidemic, and anti-diabetic).

**Supplemental Table 6.** Association of baseline TyG-BRI with new-onset CVD in UK Biobank and CHARLS

| Variable | Case, n (%) | Model 1^a^ | | Model 2^b^ | | Model 3^c^ | |
| --- | --- | --- | --- | --- | --- | --- | --- |
|  |  | HR (95CI) | *P* value | HR (95CI) | *P* value | HR (95CI) | *P* value |
| **UK Biobank, N=418,510**  **CHD** | | | | | | | |
| Q 1 (<20.88) | 971(0.93) | 1.00(Ref) |  | 1.00(Ref) |  | 1.00(Ref) |  |
| Q 2 (20.88-28.03) | 1,943(1.85) | 1.44(1.34-1.56) | <0.001 | 1.41(1.30-1.52) | <0.001 | 1.40(1.29-1.52) | <0.001 |
| Q 3 (28.03-36.45) | 2,856(2.73) | 1.86(1.72-2.00) | <0.001 | 1.75(1.62-1.89) | <0.001 | 1.72(1.57-1.88) | <0.001 |
| Q 4 (≥36.45) | 3,724(3.56) | 2.43(2.26-2.61) | <0.001 | 2.18(2.03-2.35) | <0.001 | 2.05(1.87-2.25) | <0.001 |
| *P* for trend |  |  | <0.001 |  | <0.001 |  | <0.001 |
| **Stroke** | | | | | | | |
| Q 1 (<26.85) | 1,780(1.70) | 1.00(Ref) |  | 1.00(Ref) |  | 1.00(Ref) |  |
| Q 2 (26.85-34.58) | 2,542(2.43) | 1.10(1.04-1.17) | 0.002 | 1.08(1.02-1.15) | 0.014 | 1.10(1.03-1.18) | 0.004 |
| Q 3 (34.58-44.46) | 3,025(2.89) | 1.17(1.10-1.24) | <0.001 | 1.11(1.04-1.18) | <0.001 | 1.14(1.05-1.23) | <0.001 |
| Q 4 (≥44.46) | 4,081(3.90) | 1.55(1.47-1.65) | <0.001 | 1.39(1.31-.147) | <0.001 | 1.37(1.26-1.48) | <0.001 |
| *P* for trend |  |  | <0.001 |  | <0.001 |  | <0.001 |
| **AF** |  |  |  |  |  |  |  |
| Q 1 (<26.85) | 2,870(2.74) | 1.00(Ref) |  | 1.00(Ref) |  | 1.00(Ref) |  |
| Q 2 (26.85-34.58) | 4,336(4.15) | 1.09(1.03-1.14) | <0.001 | 1.08(1.03-1.13) | 0.002 | 0.94(0.89-1.11) | 0.118 |
| Q 3 (34.58-44.46) | 5,396(5.16) | 1.17(1.12-1.23) | <0.001 | 1.15(1.10-1.20) | <0.001 | 1.04(0.96-1.23) | 0.231 |
| Q 4 (≥44.46) | 7,821(7.47) | 1.70(1.63-1.78) | <0.001 | 1.63(1.56-1.70) | <0.001 | 1.16(1.09-1.23) | <0.001 |
| *P* for trend |  |  | <0.001 |  | <0.001 |  | <0.001 |
| **HF** |  |  |  |  |  |  |  |
| Q 1 (<26.85) | 1,171(1.12) | 1.00(Ref) |  | 1.00(Ref) |  | 1.00(Ref) |  |
| Q 2 (26.85-34.58) | 2,014(1.93) | 1.25(1.16-1.35) | <0.001 | 1.22(1.13-1.31) | <0.001 | 1.13(1.05-1.23) | 0.002 |
| Q 3 (34.58-44.46) | 2,922(2.79) | 1.58(1.48-1.70) | <0.001 | 1.48(1.38-1.59) | <0.001 | 1.28(1.17-1.39) | <0.001 |
| Q 4 (≥44.46) | 5,514(5.27) | 2.97(2.78-3.17) | <0.001 | 2.59(2.43-2.76) | <0.001 | 2.03(1.86-2.21) | <0.001 |
| *P* for trend |  |  | <0.001 |  | <0.001 |  | <0.001 |
| **CHARLS, N=7,923** |  |  |  |  |  |  |  |
| **Stroke** |  |  |  |  |  |  |  |
| Q 1 (<26.85) | 91(4.59) | 1.00(Ref) |  | 1.00(Ref) |  | 1.00(Ref) |  |
| Q 2 (26.85-34.58) | 128(6.47) | 1.50(1.15-1.96) | <0.001 | 1.53(1.17-2.00) | 0.002 | 1.44(1.09-1.89) | 0.010 |
| Q 3 (34.58-44.46) | 180(9.90) | 2.21(1.71-2.85) | <0.001 | 2.28(1.76-2.95) | <0.001 | 2.03(1.54-2.69) | <0.001 |
| Q 4 (≥44.46) | 227(11.46) | 2.90(2.25-3.74) | <0.001 | 3.03(2.35-3.92) | <0.001 | 2.47(1.79-3.42) | <0.001 |
| *P* for trend |  |  | <0.001 |  | <0.001 |  | <0.001 |
| **Cardiac events** |  |  |  |  |  |  |  |
| Q 1 (<26.85) | 270(13.62) | 1.00(Ref) |  | 1.00(Ref) |  | 1.00(Ref) |  |
| Q 2 (26.85-34.58) | 318(16.07) | 1.17(0.99-1.37) | 0.081 | 1.16(0.99-1.37) | <0.001 | 1.17(0.99-1.34) | 0.065 |
| Q 3 (34.58-44.46) | 352(17.77) | 1.24(1.06-1.46) | <0.001 | 1.24(1.06-1.46) | <0.001 | 1.22(1.03-1.41) | 0.008 |
| Q 4 (≥44.46) | 451(22.78) | 1.56(1.33-1.83) | <0.001 | 1.56(1.33-1.83) | <0.001 | 1.52(1.28-1.85) | <0.001 |
| *P* for trend |  |  | <0.001 |  | <0.001 |  | <0.001 |

^a^ Model 1 adjusted for age, sex;

^b^ Model 2 further adjusted for region, education level, smoking status, drinking status based on model 1;

^c^ Model 3 further adjusted for dyslipidemia, systolic blood pressure, history of CKD, physical activity, socioeconomic status and medications (anti-hypertensive, anti-dyslipidemic, and anti-diabetic) based on model 2.

**Supplemental Table 7.** Subgroup analysis of HRs (95% CI) of TyG-BRI classes with new-onset CVD in CHARLS and UK Biobank.

| Characteristics | Class 1 | Class 2 | Class 3 | Class 4 | Class 5 | *P* value | *P-_interaction_* |
| --- | --- | --- | --- | --- | --- | --- | --- |
| **UK Biobank, N=11,682** | | | | | | | |
| Sex |  |  |  |  |  |  |  |
| Male | 1.00(Ref) | 0.95(0.77-1.17) | 1.27(1.00-1.60) | 1.41(1.05-1.89) | 2.67(1.76-4.04) | <0.001 | <0.001 |
| Female | 1.00(Ref) | 1.01(0.77-1.31) | 1.14(0.82-1.59) | 1.68(1.15-2.46) | 2.41(1.40-4.15) | <0.001 |  |
| Age, years |  |  |  |  |  |  |  |
| <60 | 1.00(Ref) | 1.13(0.84-1.51) | 1.52(1.08-2.13) | 1.83(1.20-2.78) | 2.24(1.26-3.98) | <0.001 | <0.001 |
| ≥60 | 1.00(Ref) | 0.90(0.74-1.10) | 1.14(0.91-1.43) | 1.35(1.02-1.78) | 2.80(1.86-4.21) | <0.001 |  |
| Smoking |  |  |  |  |  |  |  |
| No | 1.00(Ref) | 0.98(0.79-1.22) | 1.23(0.87-1.75) | 1.32(1.02-1.70) | 2.54(1.55-4.15) | <0.001 | <0.001 |
| Yes | 1.00(Ref) | 0.97(0.74-1.27) | 1.60(1.12-2.29) | 1.60(1.12-2.29) | 2.98(1.85-4.80) | <0.001 |  |
| Drinking |  |  |  |  |  |  |  |
| No | 1.00(Ref) | 2.30(0.81-6.49) | 3.01(0.85-10.67) | 3.38(1.09-10.48) | 5.81(1.17-28.70) | <0.001 | <0.001 |
| Yes | 1.00(Ref) | 0.93(0.79-1.10) | 1.24(1.02-1.50) | 1.43(1.13-1.83) | 2.54(1.80-3.57) | <0.001 |  |
| BMI, kg/m^2^ |  |  |  |  |  |  |  |
| <25 | 1.00(Ref) | 0.77(0.43-1.36) | 3.30(1.36-8.02) | 4.07(2.50-6.63) | 4.07(2.50-6.63) | <0.001 | <0.001 |
| ≥25 | 1.00(Ref) | 0.66(0.46-0.93) | 2.35(1.67-3.30) | 2.88(2.07-4.02) | 2.88(2.07-4.02) | <0.001 |  |
| **CHARLS, N=4,637** | | | | | | | |
| Sex |  |  |  |  |  |  |  |
| Male | 1.00(Ref) | 1.20(0.87-1.67) | 1.36(1.08-1.89) | 1.61(1.10-2.36) | 1.87(1.35-2.61) | <0.001 | 0.042 |
| Female | 1.00(Ref) | 1.21(0.87-1.71) | 1.59(1.18-2.14) | 1.80(1.31-2.48) | 2.19(1.57-3.04) | <0.001 |  |
| Age, years |  |  |  |  |  |  |  |
| <60 | 1.00(Ref) | 1.16(0.85-1.59) | 1.29(0.94-1.78) | 1.48(1.06-2.08) | 1.80(1.37-2.39) | <0.001 | 0.879 |
| ≥60 | 1.00(Ref) | 1.34(0.94-1.90) | 1.44(1.02-1.98) | 1.59(1.14-2.22) | 1.89(1.22-2.82) | <0.001 |  |
| Smoking |  |  |  |  |  |  |  |
| No | 1.00(Ref) | 1.18(0.86-1.63) | 1.38(1.00-1.90) | 1.65(1.22-2.24) | 1.88(1.43-2.51) | <0.001 | 0.136 |
| Yes | 1.00(Ref) | 1.25(0.86-1.80) | 1.32(0.91-2.86) | 1.42(1.03-2.03) | 1.78(1.14-2.76) | <0.001 |  |
| Drinking |  |  |  |  |  |  |  |
| No | 1.00(Ref) | 1.29(0.94-1.77) | 1.39(1.00-1.93) | 1.61(1.19-2.17) | 1.75(1.17-2.64) | <0.001 | 0.430 |
| Yes | 1.00(Ref) | 1.19(0.82-1.71) | 1.26(0.88-1.81) | 1.40(1.11-2.71) | 1.59(1.05-2.40) | 0.031 |  |
| BMI, kg/m^2^ |  |  |  |  |  |  |  |
| <24 | 1.00(Ref) | 1.36(1.08-1.71) | 1.58(1.27-1.97) | 1.65(1.33-2.05) | 2.09(1.58-2.76) | <0.001 | 0.018 |
| ≥24 | 1.00(Ref) | 1.21(0.32-4.60) | 2.02(0.73-5.64) | 2.49(0.93-6.68) | 3.16(1.09-9.19) | <0.001 |  |

In the multivariate models, confounding factors such as age, sex, region, education level, smoking status, drinking status, dyslipidemia, systolic blood pressure, history of CKD, physical activity, socioeconomic status and medications (anti-hypertensive, anti-dyslipidemic, and anti-diabetic).

**Supplemental Table 8.** Association of different TyG-BRI classes with new-onset CVD in UK Biobank and CHARLS

| Variable | Case, n (%) | Model 1^a^ | | Model 2^b^ | | Model 3^c^ | |
| --- | --- | --- | --- | --- | --- | --- | --- |
|  |  | HR (95CI) | *P* value | HR (95CI) | *P* value | HR (95CI) | *P* value |
| **UK Biobank, N=11,682**  **CHD** | | | | | | | |
| Class 1 | 52(1.02) | 1.00(Ref) |  | 1.00(Ref) |  | 1.00(Ref) |  |
| Class 2 | 90(1.97) | 1.40(0.99-1.98) | 0.061 | 1.34(0.94-1.89) | 0.104 | 1.22(0.85-1.73) | 0.280 |
| Class 3 | 61(2.89) | 1.93(1.32-2.81) | <0.001 | 1.76(1.20-2.58) | 0.004 | 1.50(1.02-2.21) | 0.028 |
| Class 4 | 23(2.49) | 1.71(1.04-2.80) | 0.035 | 1.50(0.91-2.47) | 0.115 | 1.24(0.74-2.06) | 0.360 |
| Class 5 | 16(5.67) | 4.27(2.43-7.49) | <0.001 | 3.57(2.02-6.33) | <0.001 | 2.72(1.51-4.90) | <0.001 |
| *P* for trend |  |  | <0.001 |  | <0.001 |  | <0.001 |
| **Stroke** | | | | | | | |
| Class 1 | 80(1.57) | 1.00(Ref) |  | 1.00(Ref) |  | 1.00(Ref) |  |
| Class 2 | 85(1.86) | 0.93(0.68-1.27) | 0.656 | 0.90(0.66-1.23) | 0.504 | 0.88(0.64-1.21) | 0.443 |
| Class 3 | 58(2.75) | 1.29(0.92-1.83) | 0.145 | 1.21(0.85-1.71) | 0.292 | 1.18(0.83-1.69) | 0.365 |
| Class 4 | 40(4.33) | 2.06(1.40-3.03) | <0.001 | 1.84(1.25-2.73) | 0.002 | 1.80(1.20-2.69) | 0.004 |
| Class 5 | 10(3.55) | 1.81(0.94-3.51) | 0.077 | 1.53(0.79-2.98) | 0.211 | 1.49(0.75-2.93) | 0.255 |
| *P* for trend |  |  | <0.001 |  | 0.002 |  | 0.125 |
| **AF** |  |  |  |  |  |  |  |
| Class 1 | 192(3.77) | 1.00(Ref) |  | 1.00(Ref) |  | 1.00(Ref) |  |
| Class 2 | 210(4.60) | 0.89(0.73-1.08) | 0.236 | 0.88(0.72-1.08) | 0.216 | 0.84(0.68-1.02) | 0.083 |
| Class 3 | 153(7.26) | 1.32(1.06-1.64) | 0.013 | 1.31(1.05-1.63) | 0.017 | 1.19(0.95-1.49) | 0.131 |
| Class 4 | 82(8.88) | 1.65(1.27-2.14) | <0.001 | 1.63(1.25-2.12) | <0.001 | 1.42(1.09-1.87) | 0.011 |
| Class 5 | 41(14.54) | 3.06(2.18-4.29) | <0.001 | 2.99(2.12-4.22) | <0.001 | 2.45(1.72-3.40) | <0.001 |
| *P* for trend |  |  | <0.001 |  | <0.001 |  | <0.001 |
| **HF** |  |  |  |  |  |  |  |
| Class 1 | 60(1.18) | 1.00(Ref) |  | 1.00(Ref) |  |  |  |
| Class 2 | 92(2.01) | 1.28(0.92-1.78) | 0.147 | 1.22(0.87-1.70) | 0.250 | 1.10(0.78-1.53) | 0.597 |
| Class 3 | 56(2.66) | 1.56(1.07-2.26) | 0.020 | 1.41(0.97-2.05) | 0.073 | 1.19(0.81-1.74) | 0.383 |
| Class 4 | 38(4.12) | 2.43(1.61-3.66) | <0.001 | 2.12(1.40-3.22) | <0.001 | 1.70(1.11-2.61) | 0.015 |
| Class 5 | 27(9.57) | 6.45(4.08-9.19) | <0.001 | 5.38(3.38-8.57) | <0.001 | 3.91(2.41-6.34) | <0.001 |
| *P* for trend |  |  | <0.001 |  | <0.001 |  | <0.001 |
| **CHARLS, N=4,637** |  |  |  |  |  |  |  |
| **Stroke** |  |  |  |  |  |  |  |
| Class 1 | 67(4.28) | 1.00(Ref) |  | 1.00(Ref) |  | 1.00(Ref) |  |
| Class 2 | 56(6.67) | 1.75(1.22-2.50) | <0.001 | 1.77(1.23-2.54) | 0.002 | 1.60(1.10-2.33) | 0.014 |
| Class 3 | 68(7.87) | 1.94(1.38-2.73) | <0.001 | 1.99(1.42-2.80) | <0.001 | 1.78(1.24-2.56) | 0.002 |
| Class 4 | 89(8.94) | 2.38(1.71-3.30) | <0.001 | 2.45(1.76-3.41) | <0.001 | 1.99(1.34-2.97) | <0.001 |
| Class 5 | 39(10.51) | 2.90(1.91-4.41) | <0.001 | 3.01(1.97-4.58) | <0.001 | 2.21(1.29-3.79) | <0.001 |
| *P* for trend |  |  | <0.001 |  | <0.001 |  | <0.001 |
| **Cardiac events** |  |  |  |  |  |  |  |
| Class 1 | 138(8.81) | 1.00(Ref) |  | 1.00(Ref) |  | 1.00(Ref) |  |
| Class 2 | 86(10.24) | 1.09(0.83-1.44) | 0.530 | 1.10(0.83-1.44) | 0.505 | 1.08(0.75-1.33) | 0.881 |
| Class 3 | 111(12.85) | 1.39(1.08-1.79) | 0.010 | 1.40(1.09-1.81) | 0.009 | 1.26(1.06-1.65) | 0.045 |
| Class 4 | 135(13.57) | 1.41(1.10-1.80) | 0.006 | 1.42(1.11-1.83) | 0.005 | 1.31(1.07-1.58) | 0.036 |
| Class 5 | 66(17.79) | 1.79(1.32-2.44) | <0.001 | 1.82(1.33-2.48) | <0.001 | 1.45(1.19-2.31) | 0.011 |
| *P* for trend |  |  | <0.001 |  | <0.001 |  | 0.015 |

^a^ Model 1 adjusted for age, sex;

^b^ Model 2 further adjusted for region, education level, smoking status, drinking status based on model 1;

^c^ Model 3 further adjusted for dyslipidemia, systolic blood pressure, history of CKD, physical activity, socioeconomic status and medications (anti-hypertensive, anti-dyslipidemic, and anti-diabetic) based on model 2.

**Supplemental Figure 1.** Flow chart of sample selection and the exclusion criteria in UK Biobank.

**Supplemental Figure 2.** Flow chart of sample selection and the exclusion criteria in CHARLS.

**Supplemental Figure 3.** Association of baseline TyG-BRI with risk of CHD, stroke, AF and HF in UK Biobank.

Hazard ratios and 95% CIs derived from restricted cubic spline regression, with knots placed at the 5th, 35th, 65th, and 95th percentiles of the distribution of baseline TyG-BRI. The reference point for TyG-BRI is the midpoint (16.75) of the reference group from categorical analysis. The lowest 1% and highest 1% of participants were not shown in the figures for small sample sizes. Hazard ratios were adjusted for the same variables as multivariable-adjusted model in Table 1.

**Supplemental Figure 4.** Association of baseline TyG-BRI with risk of stroke and cardiac events in CHARLS.

Hazard ratios and 95% CIs derived from restricted cubic spline regression, with knots placed at the 5th, 35th, 65th, and 95th percentiles of the distribution of baseline TyG-BRI. The reference point for TyG-BRI is the midpoint (22.65) of the reference group from categorical analysis. The lowest 1% and highest 1% of participants were not shown in the figures for small sample sizes. Hazard ratios were adjusted for the same variables as multivariable-adjusted model in Table 1.

**Supplemental Figure 5.** Kaplan–Meier curves for the cumulative risk of CVD risk according to different TyG-BRI classes in UK Biobank (A: CHD; B: Stroke; C: AF; D: HF).

**Supplemental Figure 6.** Kaplan–Meier curves for the cumulative risk of CVD risk according to different TyG-BRI classes in CHARLS (A: Stroke; B: Cardiac events).
