# Supplementary material for: Associations of the Baseline Level and Change in Triglyceride Glucose‐Body Roundness Index With Cardiovascular Diseases: Two Large Prospective Cohorts in Europe and Asia
Source: Kaohsiung J Med Sci. 2026 Oct 22:e70305. Online ahead of print. doi: 10.1002/kjm2.70305 (PMC13688557; doi:10.1002/kjm2.70305)
Supplement: Supplementary file 2 [file KJM2-9999-e70305-s002.docx]

**Supplemental Table 9.** Subgroup analysis of HRs (95% CI) of TyG-BRI classes with new-onset CVD in CHARLS and UK Biobank.

| Characteristics | Class 1 | Class 2 | Class 3 | Class 4 | Class 5 | *P* value | *P-_interaction_* |
| --- | --- | --- | --- | --- | --- | --- | --- |
| **UK Biobank, N=11,682** | | | | | | | |
| Sex |  |  |  |  |  |  |  |
| Male | 1.00(Ref) | 0.95(0.77-1.17) | 1.27(1.00-1.60) | 1.41(1.05-1.89) | 2.67(1.76-4.04) | <0.001 | <0.001 |
| Female | 1.00(Ref) | 1.01(0.77-1.31) | 1.14(0.82-1.59) | 1.68(1.15-2.46) | 2.41(1.40-4.15) | <0.001 |  |
| Age, years |  |  |  |  |  |  |  |
| <60 | 1.00(Ref) | 1.13(0.84-1.51) | 1.52(1.08-2.13) | 1.83(1.20-2.78) | 2.24(1.26-3.98) | <0.001 | <0.001 |
| ≥60 | 1.00(Ref) | 0.90(0.74-1.10) | 1.14(0.91-1.43) | 1.35(1.02-1.78) | 2.80(1.86-4.21) | <0.001 |  |
| Smoking |  |  |  |  |  |  |  |
| No | 1.00(Ref) | 0.98(0.79-1.22) | 1.23(0.87-1.75) | 1.32(1.02-1.70) | 2.54(1.55-4.15) | <0.001 | <0.001 |
| Yes | 1.00(Ref) | 0.97(0.74-1.27) | 1.60(1.12-2.29) | 1.60(1.12-2.29) | 2.98(1.85-4.80) | <0.001 |  |
| Drinking |  |  |  |  |  |  |  |
| No | 1.00(Ref) | 2.30(0.81-6.49) | 3.01(0.85-10.67) | 3.38(1.09-10.48) | 5.81(1.17-28.70) | <0.001 | <0.001 |
| Yes | 1.00(Ref) | 0.93(0.79-1.10) | 1.24(1.02-1.50) | 1.43(1.13-1.83) | 2.54(1.80-3.57) | <0.001 |  |
| BMI, kg/m^2^ |  |  |  |  |  |  |  |
| <25 | 1.00(Ref) | 0.77(0.43-1.36) | 3.30(1.36-8.02) | 4.07(2.50-6.63) | 4.07(2.50-6.63) | <0.001 | <0.001 |
| ≥25 | 1.00(Ref) | 0.66(0.46-0.93) | 2.35(1.67-3.30) | 2.88(2.07-4.02) | 2.88(2.07-4.02) | <0.001 |  |
| **CHARLS, N=4,637** | | | | | | | |
| Sex |  |  |  |  |  |  |  |
| Male | 1.00(Ref) | 1.40(1.04-1.90) | 1.55(1.14-2.11) | 1.92(101-3.67) | 2.13(1.58-2.89) | <0.001 | 0.042 |
| Female | 1.00(Ref) | 1.21(0.87-1.71) | 1.59(1.18-2.14) | 1.80(1.31-2.48) | 2.19(1.57-3.04) | <0.001 |  |
| Age, years |  |  |  |  |  |  |  |
| <60 | 1.00(Ref) | 1.29(0.95-1.75) | 1.44(1.07-1.95) | 1.82(1.38-2.42) | 2.11(1.45-3.05) | <0.001 | 0.879 |
| ≥60 | 1.00(Ref) | 1.43(1.02-2.00) | 1.59(1.17-2.17) | 1.76(1.30-2.38) | 2.24(1.54-3.25) | <0.001 |  |
| Smoking |  |  |  |  |  |  |  |
| No | 1.00(Ref) | 1.30(0.95-1.78) | 1.71(1.29-2.26) | 1.87(1.40-2.50) | 2.37(1.72-3.25) | <0.001 | 0.136 |
| Yes | 1.00(Ref) | 1.33(0.95-1.85) | 1.50(1.08-2.08) | 1.62(1.15-3.06) | 1.99(1.44-2.77) | <0.001 |  |
| Drinking |  |  |  |  |  |  |  |
| No | 1.00(Ref) | 1.41(1.04-1.91) | 1.67(1.26-2.21) | 1.80(1.35-2.39) | 2.35(1.70-3.25) | <0.001 | 0.430 |
| Yes | 1.00(Ref) | 1.29(0.92-1.82) | 1.42(1.02-1.96) | 1.90(1.14-3.18) | 1.94(1.41-2.66) | <0.001 |  |
| BMI, kg/m^2^ |  |  |  |  |  |  |  |
| <24 | 1.00(Ref) | 1.36(1.08-1.71) | 1.58(1.27-1.97) | 1.65(1.33-2.05) | 2.09(1.58-2.76) | <0.001 | 0.018 |
| ≥24 | 1.00(Ref) | 1.21(0.32-4.60) | 2.02(0.73-5.64) | 2.49(0.93-6.68) | 3.16(1.09-9.19) | <0.001 |  |

In the multivariate models, confounding factors such as age, sex, region, education level, smoking status, drinking status, and dyslipidemia and medications (anti-hypertensive, anti-dyslipidemic, and anti-diabetic).

**Supplemental Table 10.** Association of different TyG-BRI classes with new-onset CVD in UK Biobank and CHARLS

| Variable | Case, n (%) | Model 1^a^ | | Model 2^b^ | | Model 3^c^ | |
| --- | --- | --- | --- | --- | --- | --- | --- |
|  |  | HR (95CI) | *P* value | HR (95CI) | *P* value | HR (95CI) | *P* value |
| **UK Biobank, N=11,682**  **CHD** | | | | | | | |
| Class 1 | 52(1.02) | 1.00(Ref) |  | 1.00(Ref) |  | 1.00(Ref) |  |
| Class 2 | 90(1.97) | 1.40(0.99-1.98) | 0.061 | 1.34(0.94-1.89) | 0.104 | 1.24(0.87-1.77) | 0.228 |
| Class 3 | 61(2.89) | 1.93(1.32-2.81) | <0.001 | 1.76(1.20-2.58) | 0.004 | 1.54(1.05-2.28) | 0.028 |
| Class 4 | 23(2.49) | 1.71(1.04-2.80) | 0.035 | 1.50(0.91-2.47) | 0.115 | 1.27(0.76-2.11) | 0.360 |
| Class 5 | 16(5.67) | 4.27(2.43-7.49) | <0.001 | 3.57(2.02-6.33) | <0.001 | 2.80(1.55-5.04) | <0.001 |
| *P* for trend |  |  | <0.001 |  | <0.001 |  | <0.001 |
| **Stroke** | | | | | | | |
| Class 1 | 80(1.57) | 1.00(Ref) |  | 1.00(Ref) |  | 1.00(Ref) |  |
| Class 2 | 85(1.86) | 0.93(0.68-1.27) | 0.656 | 0.90(0.66-1.23) | 0.504 | 0.90(0.66-1.23) | 0.504 |
| Class 3 | 58(2.75) | 1.29(0.92-1.83) | 0.145 | 1.21(0.85-1.71) | 0.292 | 1.21(0.85-1.72) | 0.302 |
| Class 4 | 40(4.33) | 2.06(1.40-3.03) | <0.001 | 1.84(1.25-2.73) | 0.002 | 1.84(1.23-2.74) | 0.003 |
| Class 5 | 10(3.55) | 1.81(0.94-3.51) | 0.077 | 1.53(0.79-2.98) | 0.211 | 1.52(0.77-3.00) | 0.228 |
| *P* for trend |  |  | <0.001 |  | 0.002 |  | 0.003 |
| **AF** |  |  |  |  |  |  |  |
| Class 1 | 192(3.77) | 1.00(Ref) |  | 1.00(Ref) |  | 1.00(Ref) |  |
| Class 2 | 210(4.60) | 0.89(0.73-1.08) | 0.236 | 0.88(0.72-1.08) | 0.216 | 0.83(0.68-1.02) | 0.076 |
| Class 3 | 153(7.26) | 1.32(1.06-1.64) | 0.013 | 1.31(1.05-1.63) | 0.017 | 1.18(0.95-1.48) | 0.140 |
| Class 4 | 82(8.88) | 1.65(1.27-2.14) | <0.001 | 1.63(1.25-2.12) | <0.001 | 1.42(1.08-1.86) | 0.011 |
| Class 5 | 41(14.54) | 3.06(2.18-4.29) | <0.001 | 2.99(2.12-4.22) | <0.001 | 2.44(1.71-3.47) | <0.001 |
| *P* for trend |  |  | <0.001 |  | <0.001 |  | <0.001 |
| **HF** |  |  |  |  |  |  |  |
| Class 1 | 60(1.18) | 1.00(Ref) |  | 1.00(Ref) |  |  |  |
| Class 2 | 92(2.01) | 1.28(0.92-1.78) | 0.147 | 1.22(0.87-1.70) | 0.250 | 1.10(0.79-1.54) | 0.564 |
| Class 3 | 56(2.66) | 1.56(1.07-2.26) | 0.020 | 1.41(0.97-2.05) | 0.073 | 1.20(0.82-1.75) | 0.355 |
| Class 4 | 38(4.12) | 2.43(1.61-3.66) | <0.001 | 2.12(1.40-3.22) | <0.001 | 1.72(1.12-2.63) | 0.013 |
| Class 5 | 27(9.57) | 6.45(4.08-9.19) | <0.001 | 5.38(3.38-8.57) | <0.001 | 3.94(2.43-6.39) | <0.001 |
| *P* for trend |  |  | <0.001 |  | <0.001 |  | <0.001 |
| **CHARLS, N=4,637** |  |  |  |  |  |  |  |
| **Stroke** |  |  |  |  |  |  |  |
| Class 1 | 67(4.28) | 1.00(Ref) |  | 1.00(Ref) |  | 1.00(Ref) |  |
| Class 2 | 56(6.67) | 1.75(1.22-2.50) | <0.001 | 1.77(1.23-2.54) | 0.002 | 1.77(1.23-2.54) | 0.002 |
| Class 3 | 68(7.87) | 1.94(1.38-2.73) | <0.001 | 1.99(1.42-2.80) | <0.001 | 2.00(1.42-2.82) | <0.001 |
| Class 4 | 89(8.94) | 2.38(1.71-3.30) | <0.001 | 2.45(1.76-3.41) | <0.001 | 2.46(1.76-3.43) | <0.001 |
| Class 5 | 39(10.51) | 2.90(1.91-4.41) | <0.001 | 3.01(1.97-4.58) | <0.001 | 3.05(2.00-4.64) | <0.001 |
| *P* for trend |  |  | <0.001 |  | <0.001 |  | <0.001 |
| **Cardiac events** |  |  |  |  |  |  |  |
| Class 1 | 138(8.81) | 1.00(Ref) |  | 1.00(Ref) |  | 1.00(Ref) |  |
| Class 2 | 86(10.24) | 1.09(0.83-1.44) | 0.530 | 1.10(0.83-1.44) | 0.505 | 1.10(0.83-1.44) | 0.517 |
| Class 3 | 111(12.85) | 1.39(1.08-1.79) | 0.010 | 1.40(1.09-1.81) | 0.009 | 1.40(1.09-1.81) | 0.009 |
| Class 4 | 135(13.57) | 1.41(1.10-1.80) | 0.006 | 1.42(1.11-1.83) | 0.005 | 1.42(1.11-1.82) | 0.006 |
| Class 5 | 66(17.79) | 1.79(1.32-2.44) | <0.001 | 1.82(1.33-2.48) | <0.001 | 1.81(1.33-2.47) | <0.001 |
| *P* for trend |  |  | <0.001 |  | <0.001 |  | <0.001 |

^a^ Model 1 adjusted for age, sex;

^b^ Model 2 further adjusted for region, education level, smoking status, drinking status based on model 1;

^c^ Model 3 further adjusted for dyslipidemia and medications (anti-hypertensive, anti-dyslipidemic, and anti-diabetic) based on model 2.
